# Growing Teratoma Syndrome with Synchronous Gliomatosis Peritonei during Chemotherapy in Ovarian Immature Teratoma: A Case Report and Literature Review

**DOI:** 10.3390/curroncol29090501

**Published:** 2022-09-04

**Authors:** Sijian Li, Na Su, Congwei Jia, Xinyue Zhang, Min Yin, Jiaxin Yang

**Affiliations:** 1 National Clinical Research Center for Obstetric and Gynecologic Diseases, Department of Obstetrics and Gynecology, Peking Union Medical College Hospital, Chinese Academy of Medical Sciences, Peking Union Medical College, Beijing 100730, China; 2 Department of Ultrasound, Peking Union Medical College Hospital, Chinese Academy of Medical Sciences, Peking Union Medical College, Beijing 100730, China; 3 Department of Pathology, Peking Union Medical College Hospital, Chinese Academy of Medical Sciences, Peking Union Medical College, Beijing 100730, China

**Keywords:** ovarian tumors, immature teratoma, growing teratoma syndrome, gliomatosis peritonei, treatment

## Abstract

Coexistent growing teratoma syndrome (GTS) and gliomatosis peritonei (GP) arising during chemotherapy of ovarian immature teratoma (IMT) is extremely rare and can be misdiagnosed as recurrent or progressive disease. We present a 33-year-old woman diagnosed with GTS with synchronous GP during chemotherapy of IMT. She underwent ovarian cystectomy due to ovarian immature teratoma and chemotherapy were administered. The α-fetoprotein (AFP) concentration decreased from 28.7 ng/mL to normal after the second cycle. Four days after the third cycle of chemotherapy, ultrasound and CT revealed an 8-cm mass with negative tumor markers in the pouch of Douglas. An exploratory laparotomy was conducted, and a smooth round cystic-solid 8-cm mass was noted in the pouch of Douglas. Extensive peritoneal seeding glial nodules were also observed on the surface of the uterus, peritoneum, and omentum. The patient underwent a partial omentectomy, intact resection of the tumor, and resection of most of the glial nodules. Postoperative pathology demonstrated a pure mature cystic teratoma component in the mass, as well as diffuse GP involving the uterine serosa, peritoneum, and omentum; this diagnosis of GTS with synchorous GP should be considered in IMT patients with mass newly identified during chemotherapy while tumor markers are normal after treatment.

## 1. Introduction

Ovarian immature teratoma (IMT) is one of the most common histologic subtypes of malignant ovarian germ cell tumors that contain tissue from all three germ layers as well as immature neural components, representing approximately one-third of cases [1,2]. When IMT tumors expand after surgery and/or chemotherapy without elevated tumor markers or immature elements, they are characterized as “growing teratoma syndrome (GTS)”, which accounts for between 13% and 20% of IMT patients [3,4,5]. Gliomatosis peritonei (GP) is an extremely rare entity defined as peritoneal and omental seeding of mature glial tissue, with only about 100 cases documented in English literature [6,7].

GTS and GP often occur after initial surgery or chemotherapy but can sometimes develop during chemotherapy [8], which may confuse physicians and lead to a misdiagnosis of disease recurrence and/or treatment failure; moreover, coexisting GTS and GP has rarely been reported, and there is little data on its clinical characteristics. To further understand the characteristics and outcomes in patients with concomitant GTS and GP, we present the case of a patient with synchronous GTS and GP identified during chemotherapy and review the relevant literature.

## 2. Case

**Figure 1:**
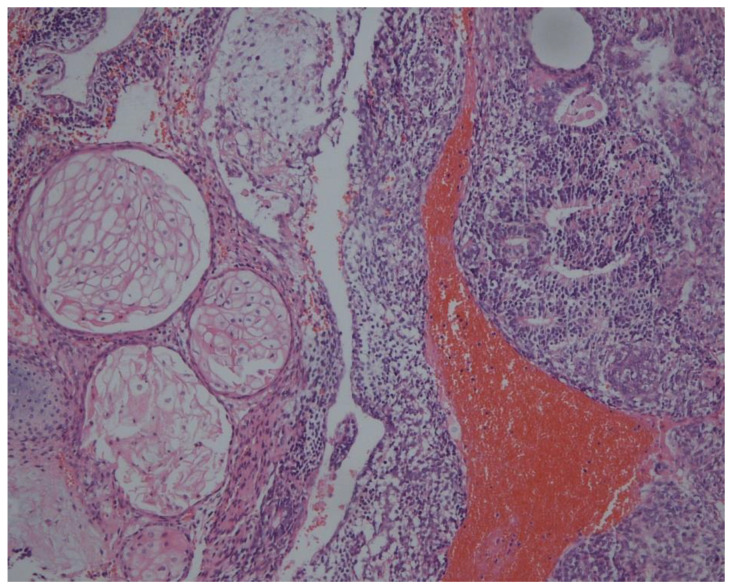
Paraffin pathology confirmed that most of the right ovarian tumor was composed of mature teratoma with focal primitive neuroepithelial elements (HE staining, 20×).

A 33-year-old woman at 34 weeks gestation presented to the Xuanwu hospital emergency department with severe abdominal pain on the end of November 2021. 5-cm bilateral ovarian teratomas had been noted in May 2021, but she had no further interventions as she was in her first trimester at the time. A diagnosis of ruptured ovarian teratoma was established, and she had a cesarean section with bilateral ovarian cystectomy. The cesarean section was conducted to avoid second surgery within nearly 6 weeks and potential postoperative treatment may affect the fetus. She delivered a healthy female baby of 2360 g. Intraoperative exploration showed a 25 × 25 × 20 cm multilocular tumor with a dominant solid component in the right ovary, along with a 7-cm cystic mass arising from the left ovary; moreover, the tumor in the right ovary was ruptured and actively bleeding. Fast frozen pathology of the right ovarian tumor revealed no malignant components, and the left ovarian cyst also ruptured during resection. The estimated blood loss was 1000 mL, and she was transfused 2 U of red blood cells. Paraffin pathology confirmed that most of the right ovarian tumor was composed of mature teratoma with focal primitive neuroepithelial elements (Figure 1), consistent with a WHO grade II immature teratoma with extensive hemorrhage and necrosis; however, the left ovarian tumor contained only mature cystic teratoma tissue, and peritoneal washing showed no tumor cells.

**Figure 2:**
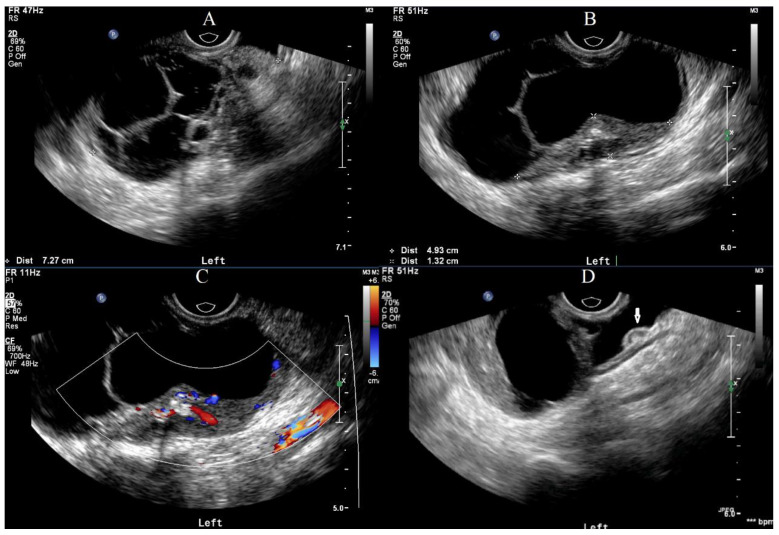
Ultrasonic features of the mass. (**A**) A multilocular solid-cystic mass with a clear boundary was seen in the left adnexal area, in which lipid and hair-like hyper-echo mass was also observed. (**B**) Calcification can be seen in solid hypo-echo of the mass. (**C**) The CDFI revealed scattered, short strips of blood flow signals in the solid proportion of this mass. (**D**) Implant nodules can be seen on the intestinal surface.

**Figure 3:**
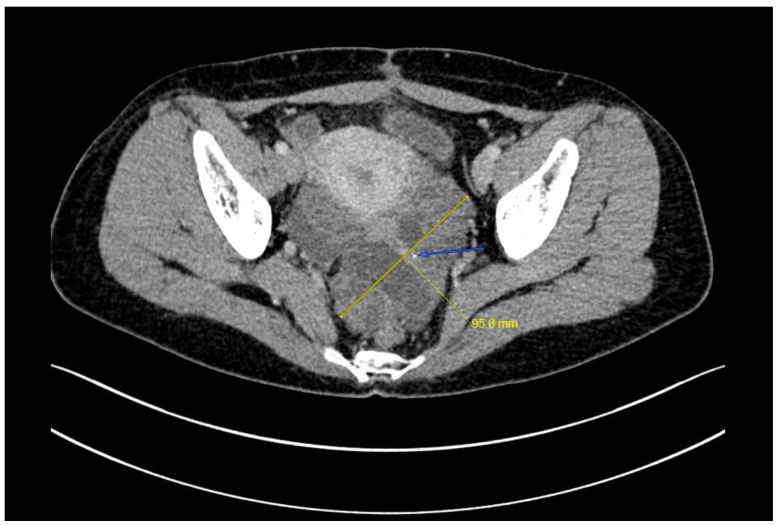
A 9.5 × 6.2 cm mass was noted left posterior of the uterus with an enhancement of solid component but not cystic component, no enlarged retroperitoneal lymph node was noted (arrow, calcification).

A right ovarian immature teratoma, grade 2, stage IC2 was diagnosed based on the FIGO stage classification for ovarian tumors. A chemotherapy regimen including bleomycin, etoposide, and cisplatin (BEP) was given for three cycles based on the NCCN clinical guidelines for ovarian germ cell tumors. AFP levels quickly decreased from 27.8 ng/mL to normal after two cycles of chemotherapy, and the last cycle was completed in mid-March 2022. The serum CA19-9 was less than 2 U/mL before surgery. Repeat ultrasonography conducted one month after the surgery did not show any abnormalities, but an ultrasound examination on five days after the last cycle of chemotherapy showed an approximately 8-cm solid cystic mass located in the left posterior uterus. The patient was then referred to our hospital. A repeat blood draw showed that serum AFP, CA125, NSE, and CA19-9 were all within normal ranges. Recurrence of immature teratoma and progressive disease could not be excluded. Further ultrasonography conducted by an expert demonstrated an 8.0 × 7.3 cm mixed-echo, lobulated mass with clear boundaries in the left adnexal area. The presence of lipid and hair-like echo, as well as a CDFI that revealed only scattered, short strips of blood flow signals in a small portion of the mass, were suggestive of GTS (Figure 2). Enhanced abdominal-pelvic CT was also used to determine the diagnosis and showed a comparable result to the ultrasound (i.e., a solid 8-cm cystic mass with undetermined malignancy), and no enlarged retroperitoneal lymph node was observed (Figure 3).

**Figure 4:**
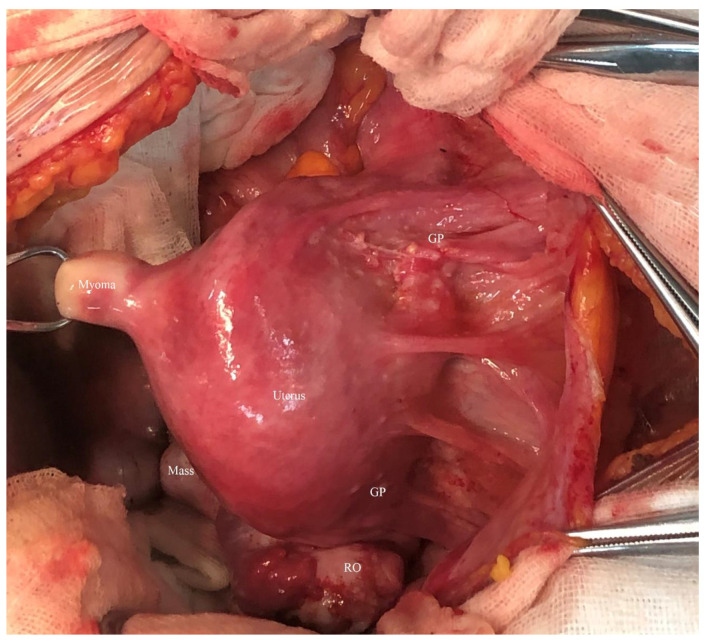
Intraoperative views during exploratory laparotomy demonstrated a small myoma located at the posterior uterine wall, and multiple glial tissue (“GP”) seeding was noted at the uterine surface, vesical peritoneal reflection. The mass was located in the pouch of Douglas, with most proportion obscured by the uterus (RO, right ovary).

**Figure 5:**
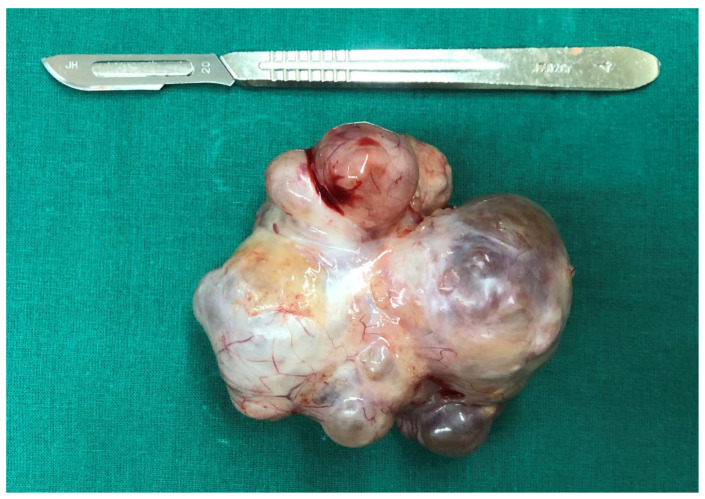
The tumor showed a smooth and irregularly lobulated gross appearance.

**Figure 6:**
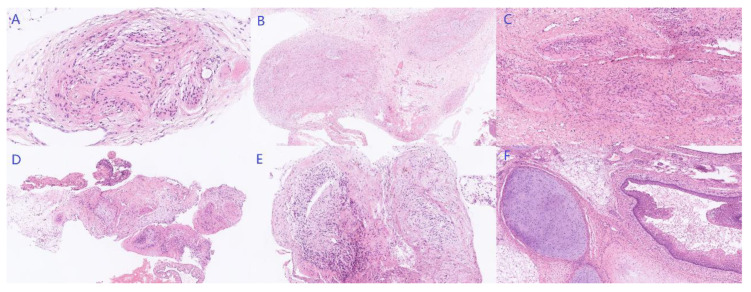
Pathology results of peritoneal seeding of GP and GTS (HE staining). Mature neuroglia presented with distinctive rounded, or ovoid nodules with well-defined boundaries involving omentum majus ((**A**), 200×), vesical peritoneal reflection ((**B**), 25×), peritoneum of ascending colon ((**C**), 50×), right ovarian cyst ((**D**), 50×), and posterior uterine wall ((**E**), 50×), while mature cartilage and squamous epithelium differentiation were observed in the mass ((**F**), 50×).

A suspected diagnosis of GTS was established, and an exploratory laparotomy was performed on April 2022. An 8-cm solid cystic mass with a smooth appearance and multiple nodular protuberances originating from the pouch of Douglas was found during the operation. There were no abnormalities in either adnexa, except for a 1-cm endometrioma on the right ovary, and there was no connection between the bilateral ovaries and the pelvic mass. There was extensive seeding of glial-like nodules in the uterine serosa, vesical peritoneal reflection, right pelvic wall peritoneum, and partial omentum majus (Figure 4). Intact mass removal, partial uterine serosa and peritoneal resection, and partial omentectomy were conducted. The tumor showed a smooth and irregularly lobulated gross appearance (Figure 5). The cross-sectional profile of the mass showed lipids and multiple calcified nodules without necrosis (Figure S1). Postoperative paraffin pathology showed a pure mature cystic teratoma in the pelvic mass without any immature components, and glioma was noted in the other resection samples, confirming the diagnosis of GTS with synchronous GP (Figure 6).

The patient recovered uneventfully and was discharged three days after her operation. Comprehensive explanations about her disease condition and a further follow-up plan were made at subsequent consultations. No further adjuvant therapy was administrated, and an ultrasound was shown to be negative four months after the operation. We recommended repeating imaging evaluations, including ultrasonography and tumor maker monitoring, every three to six months.

## 3. Discussion

GTS and GP are two distinct entities that can occur following ovarian IMT. Current research suggests that they are both benign diseases even though they have metastatic growth potential, but the mechanism underlying their development remains unclear.

Researchers have proposed two theories about the formation of GTS. The first is that chemotherapy induces a change of immature teratoma cells into mature teratoma cells. The other hypothesis suggests that mature teratoma cells survive while immature cells die following the administration of chemotherapy drugs [9]. Published theories about the development of GP are not similar to those about the development of GTS. Many previous studies suggest GP develops by spreading through capsular defects and disseminating via glial tissue angiolymphatic systems [10,11]. Other researchers propose that multipotent peritoneal stem cells may differentiate into mature glial cells under the influence of substances that are secreted by teratomas [12]; this theory suggests that ovarian teratoma and GP cells have divergent origins, an idea which is supported by molecular analyses (including polymorphic microsatellite loci), which have shown that GP cells are actually genetically unrelated to teratomas [13,14]. The different mechanisms underlying GTS and GP formation may explain their divergent growth patterns and site distributions, which can help us understand their distinct biological and clinical characteristics.

Previous studies have rarely presented detailed clinical characteristics of patients with synchronous GTS and GP [15]. Małgorzata et al. reported that a 15-year-old girl developed coexistent GTS and GP five months after the initial IMT surgery [15]. Currently, the mean time between IMT and GTS diagnosis was 7 and 18.5 months in two large cohorts, respectively [3,4]. Nonetheless, the interval between IMT and GP remains unknown. We present another case of GTS with GP that arose 3 months postoperatively during chemotherapy; this is a much earlier timeframe than what has been reported in other cases. Thus, physicians should be aware that this condition can develop rapidly.

The incidence of GTS has ranged from 13% to 40% in previous reports [3,4,5,16]; this incidence is more common than expected, suggesting clinicians should always be aware of the GTS diagnosis in patients with reappearing masses following treatment of ovarian IMT. Ultrasonography, CT, and MRI play important roles in identifying GTS because they can reveal the presence of cystic features and/or lipid and calcification components; however, the sensitivity of FDG-PET for detecting GTS is limited, with scarce data on FDG uptake [17]. Tumor markers (such as α-fetoprotein and AFP) may enhance the diagnostic capacity that elevated tumor makers usually indicate the recurrence of IMT rather than GTS, especially in patients who had normal tumor maker levels initially following treatment [18]. In 35 patients with GTS, only three had elevated tumor markers (one with elevated CA125, and two with elevated AFP) [8]; however, data on the efficacy of radiological features are limited for GP, especially because the multiple small miliary nodules may be neglected and difficult to distinguish from peritoneal carcinogenesis and tuberculosis. Indeed, published studies show that most GP cases are diagnosed intraoperatively and confirmed by pathology [6,7]. Our study was consistent with these results, in that GTS was suspected preoperatively and GP was diagnosed postoperatively. Thus, a combination of tumor markers and imaging examination can help detect GTS but not GP, and pathological analysis is necessary to confirm the diagnosis of both conditions.

GTS and GP may present simultaneously or on their own, but either way, they usually have different clinical manifestations and are subject to different treatment principles [3,6]. GTS can continue to grow and may lead to compressive symptoms and/or bowel perforation or ischemia, which can be lethal [19,20]. In a longitudinal study (mean follow-up time: 6 years) of 38 GTS patients, Bentivegna et al. showed that only one patient died of pulmonary embolism with intestinal obstruction [4]; this study also suggested that optimal debulking surgery similar to the one used in epithelial cancer could be used for GTS, and that fertility-sparing surgery should be conducted when possible. Likewise, Wang et al. found that fertility-sparing surgery was not associated with the recurrence of GTS. Among 27 patients with fertility-preserving surgeries, four patients had five successful spontaneous pregnancies [3]. Even though residual GTS disease is an independent risk factor for GTS recurrence, survival outcomes after recurrence remain excellent in this population [3,4]; however, unlike GTS, GP lesions tend to be extensive and can sometimes not be completely resected. Two large series studies enrolled 8 and 10 patients with GP and showed that all survived without complications. An additional review of the literature demonstrated a crude survival rate of 89.6% in 67 patients with GP [6,7]. Furthermore, residual peritoneal GP disease can be completely asymptomatic and stable over long periods of time [6]. Hence, the cornerstone of GTS treatment is surgical tumor removal with the aim of optimal debulking, while more conservative surgical options are recommended in patients with GP [4,6,20].

Long-term close monitoring even after GTS diagnoses should be emphasized due to the potential possibility of malignant transformation in unresected GTS. Although rare, sarcoma, carcinoid, adenocarcinoma, and primitive neuroectodermal tumor (PNET) have previously been described as malignant transformation patterns in GTS [21,22,23]. Older age and larger tumor size are associated with an increased risk of malignant transformation [24]. The interval between initial diagnosis of GTS and subsequent cancerization varied greatly, ranging from 2 years to 10 years; moreover, Wang et al. reported carcinoid transformation occurring 18 years after initial surgery in a patient with GTS [3]. Survival outcomes in both GTS and GP patients are generally excellent, but the clinical characteristics of patients with co-occurring GTS and GP have not been well defined. Further research especially focusing on long-term outcomes is needed on this rare combinatorial condition.

## 4. Conclusions

GTS with synchronous GP in ovarian IMT is an extremely rare entity that may be misdiagnosed as disease recurrence or a treatment insensitivity when it arises during postoperative chemotherapy. A suspected diagnosis should be considered when IMT patients with normalized tumor markers after treatment but reveals a newly identified mass.

## Data Availability

All data generated or analyzed during this study are included in this published article and the supplementary information files. The datasets used and/or analyzed during the current study can be obtained from the corresponding author upon reasonable request.

## Supplementary Materials

The following are available online at https://www.mdpi.com/article/10.3390/curroncol29090501/s1, Figure S1: Cutting the surface of the GTS mass reveals cystic-solid structure with lipid and multifocal calcification.
