# Supplementary material for: Growing Teratoma Syndrome with Synchronous Gliomatosis Peritonei during Chemotherapy in Ovarian Immature Teratoma: A Case Report and Literature Review
Source: Curr Oncol. 2022 Sep 4;29(9):6364–72. doi: 10.3390/curroncol29090501 (PMC9497723; doi:10.3390/curroncol29090501)
Supplement: Supplementary file 1 [file curroncol-29-00501-s001.zip › curroncol-1844320-supplementary.pdf]

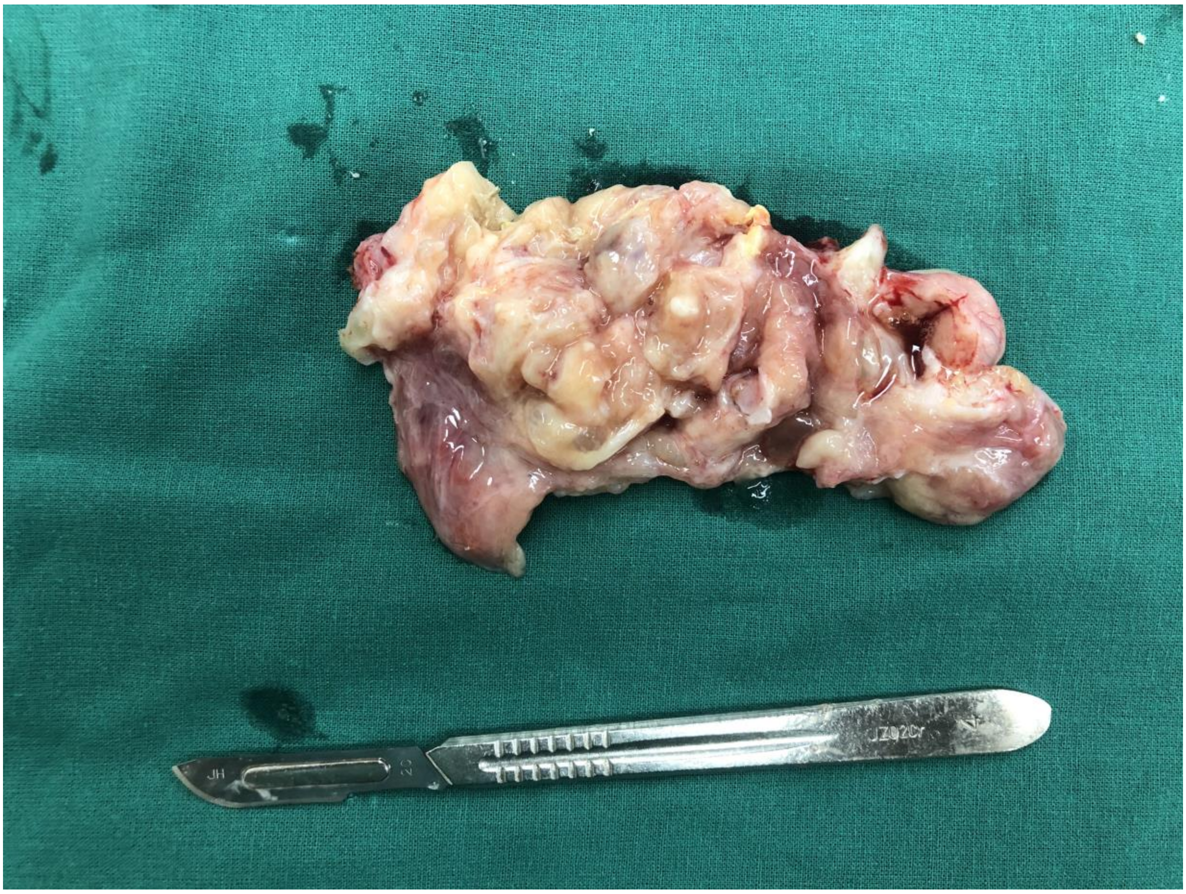

Figure S1: Cutting the surface of the GTS mass reveals cystic-solid structure with lipid and multifocal calcification.
